# Inhibition of uracil DNA glycosylase sensitizes cancer cells to 5-fluorodeoxyuridine through replication fork collapse-induced DNA damage

**DOI:** 10.18632/oncotarget.11151

**Published:** 2016-08-09

**Authors:** Yan Yan, Xiangzi Han, Yulan Qing, Allison G. Condie, Shashank Gorityala, Shuming Yang, Yan Xu, Youwei Zhang, Stanton L. Gerson

**Affiliations:** ^1^ Department of Pharmacology, Case Western Reserve University, Cleveland, OH, USA; ^2^ Department of Hematology and Oncology, Case Comprehensive Cancer Center, Case Western Reserve University, Cleveland, OH, USA; ^3^ Division of Radiopharmaceutical Science, Case Center for Imaging Research, Department of Radiology, Chemistry, and Biomedical Engineering, Case Western Reserve University, Cleveland, OH, USA; ^4^ Department of Chemistry, Cleveland State University, Cleveland, OH, USA

**Keywords:** 5-fluorodeoxyuridine, base excision repair, uracil DNA glycosylase, double strand breaks, colon cancer

## Abstract

5-fluorodeoxyuridine (5-FdU, floxuridine) is active against multiple cancers through the inhibition of thymidylate synthase, which consequently introduces uracil and 5-FU incorporation into the genome. Uracil DNA glycosylase (UDG) is one of the main enzymes responsible for the removal of uracil and 5-FU. However, how exactly UDG mediates cellular sensitivity to 5-FdU, and if so whether it is through its ability to remove uracil and 5-FU have not been well characterized. In this study, we report that UDG depletion led to incorporation of uracil and 5-FU in DNA following 5-FdU treatment and significantly enhanced 5-FdU's cytotoxicity in cancer cell lines. Co-treatment, but not post-treatment with thymidine prevented cell death of UDG depleted cells by 5-FdU, indicating that the enhanced cytotoxicity is due to the retention of uracil and 5-FU in genomic DNA in the absence of UDG. Furthermore, UDG depleted cells were arrested at late G1 and early S phase by 5-FdU, followed by accumulation of sub-G1 population indicating cell death. Mechanistically, 5-FdU dramatically reduced DNA replication speed in UDG depleted cells. UDG depletion also greatly enhanced DNA damage as shown by γH2AX foci formation. Notably, the increased γH2AX foci formation was not suppressed by caspase inhibitor treatment, suggesting that DNA damage precedes cell death induced by 5-FdU. Together, these data provide novel mechanistic insights into the roles of UDG in DNA replication, damage repair, and cell death in response to 5-FdU and suggest that UDG is a target for improving the anticancer effect of this agent.

## INTRODUCTION

Fluoropyrimidines including 5-fluorouracil (5-FU) and its deoxyribonucleoside metabolite 5-fluorodeoxyuridine (5-FdU, floxuridine) have been widely used in the treatment of various solid tumors, most notably for colorectal cancer [1–3]. Both 5-FU and 5-FdU can be converted into two forms of active metabolites in cells that disrupt DNA metabolism: fluorodeoxyuridine monophosphate (FdUMP) and fluorodeoxyuridine triphosphate (FdUTP) [4, 5]. FdUMP inhibits thymidylate synthase (TS), which consequently causes intracellular nucleotide pool imbalance with decreased dTTP and increased dUTP levels. As a result, cells will incorporate dUTP and FdUTP instead of dTTP into their DNA as the modified bases uracil and 5-FU. In addition, 5-FU can also be converted into ribonucleotide fluorouridine triphosphate (FUTP) which can then be incorporated into RNA [4, 5]. A large body of studies suggests that TS inhibition is the widely accepted mechanism by which fluropyrimidines exert their anticancer effects [1, 4–6]. Therefore, 5-FU combined with leucovorin, which specifically prolongs the duration of inhibition on TS by FdUMP, is currently considered as the standard systematic chemotherapy for advanced colorectal tumors in the clinic [7–9].

Unlike the metabolism of 5-FU into RNA, 5-FdU is primarily phosphorylated into FdUMP as a potent TS inhibitor and putatively introduces uracil and 5-FU incorporation into DNA, which therefore mainly disrupts DNA metabolism with little RNA-directed action [4–5, 10]. Additionally, 5-FdU appears to be more cytotoxic than 5-FU in a wide range of cancer cell lines and animal tumor systems [11, 12]. Although the metabolism of 5-FdU into nucleotide and DNA has been described [4, 5], it remains unclear how the DNA damage and the downstream repair pathways would impact the effectiveness of this drug. According to *in vitro* kinetic studies, base excision repair (BER) initiated by uracil DNA glycosylase (UDG) accounts for the dominant cellular activity that removes uracil and 5-FU from DNA compared with other DNA glycosylases [13]. However, whether UDG-directed BER is an effector that determines the sensitivity of TS inhibitors remains controversial. Based on studies in the yeast system [14], two models were established to explain the role of UDG in determining the cytotoxicity of TS inhibitors [5, 15]. In the first model, futile cycles of uracil and/or 5-FU incorporation and their removal by UDG lead to DNA fragmentation. One piece of evidence supporting this model showed that UDG-targeted knockdown increased the resistance to 5-FdU [16]. In the second model, accumulation of uracil and/or 5-FU in, rather than their excision from, DNA contributes to the cytotoxicity. For example, recent studies revealed that loss of UDG enhanced the cytotoxicity of cancer cells to pemetrexed and 5-FdU [17–19]. On the other hand, several studies demonstrated that overexpression or inhibition of UDG did not affect the sensitivity of TS inhibitors in human, mouse, or chicken DT40 cells [13, 20–25]. In addition, the discrepant findings have also been observed with other DNA glycosylases: SMUG1, TDG and MBD4. Enhanced sensitivity to 5-FU was reported in SMUG1 knockout murine cells due to elevated uracil and 5-FU retention [26], whereas increased resistance to 5-FU and 5-FdU was found in genetically depleted TDG or MBD4 mouse embryonic cells [27, 28].

Since UDG activity is significantly higher in colorectal tumors than in normal tissues [29], the question remains as to the role of UDG in cancer cells in response to fluoropyrimidines. In this study we investigated the impact of UDG on the sensitivity of cancer cells to 5-FdU and explored the underlying molecular mechanisms. We found that depletion of UDG induced significant accumulation of both uracil and 5-FU in genomic DNA, which indicates a prevailing role of UDG in preventing the persistence of these DNA lesions by 5-FdU treatment. Loss of UDG highly enhanced the cytotoxicity of 5-FdU. Interestingly, this increased cytotoxicity and retention of uracil and 5-FU could not be reversed by thymidine treatment after 5-FdU exposure, suggesting that the cell killing effect of 5-FdU is a result of uracil and 5-FU incorporation into DNA. UDG depleted cells were arrested at late G1 and early S phase during 5-FdU exposure; accordingly, the DNA replication speed detected by the DNA fiber assay was significantly reduced by loss of UDG, suggesting replication fork stalling or falling. Consistently, UDG depleted cells displayed sustained DNA damage following 5-FdU treatment. Collectively, these findings suggest that UDG plays an important role in the removal of uracil and 5-FU and therefore determines at least partially the therapeutic outcome of fluoropyrimidines in the clinic.

## RESULTS

### UDG removes uracil and 5-FU incorporated into DNA following 5-FdU treatment

**Figure 1:**
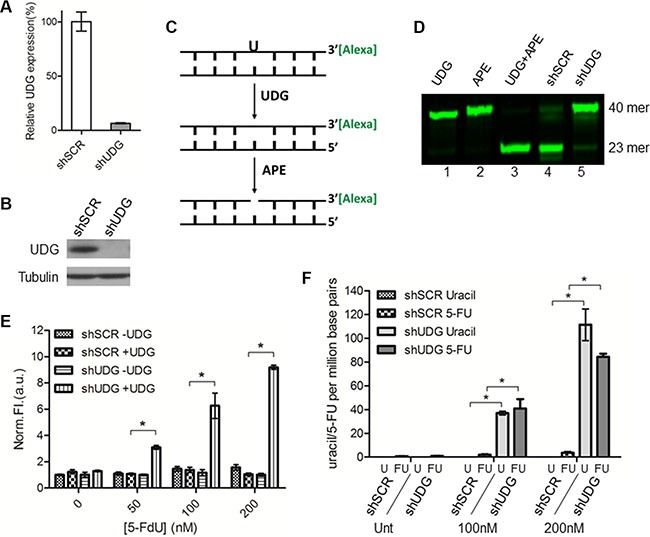
UDG depletion causes incorporation of uracil and 5-FU into genomic DNA by 5-FdU Lentiviral non-targeted scramble control shRNA (shSCR) or UDG-directed shRNA (shUDG) were transfected into DLD1 colon cancer cells, and stable cell lines were established. (**A**) UDG mRNA and (**B**) protein expression levels were determined by qPCR and western blot, respectively. The shRNA that we used targets both mitochondrial and nuclear UDG, which are collectively termed UDG in this study. (**C**) Schematic diagram of glycosylase activity assay by using 3′-Alexa tagged 40-mer DNA duplex with a uracil incorporation paired with adenine. (**D**) 10 μg nuclear extracts from DLD1 shSCR or shUDG cells were incubated with 3′-Alexa labeled oligonucleotide containing U:A base pair for 20 minutes at 37°C. Reactions with purified enzymes were used as controls. Cellular UDG activity was visualized by denaturing gel electrophoresis to separate intact 40-mer from 23-mer. (**E**) DLD1 shSCR and shUDG cells were treated with 0, 50, 100, and 200 nM 5-FdU for 48 h. Genomic DNA was extracted and treated *in vitro* with purified UDG (+ UDG) or vehicle control (− UDG). AP sites detection was performed by incubation of DNA with a cyanine-based AP site probe. Data represent mean and SD of relative fluorescence intensity normalized to 5-FdU untreated shSCR -UDG sample from three independent experiments. (*P < 0.05) (**F**) DLD1 shSCR and shUDG cells were untreated (Unt) or treated with 5-FdU 100 and 200 nM for 48 h. Genomic DNA was extracted and incubated *in vitro* with purified UDG enzyme. Uracil and 5-FU were quantified by LC-MS/MS as described in the Materials and Methods. Data represent mean and SD from three independent experiments. (*P < 0.05).

Studies have demonstrated that the nuclear form of UDG is responsible for the removal of uracil and 5-FU from DNA *in vitro* in comparison with other glycosylases [13]. To confirm this activity of UDG *in vivo*, we generated DLD1 colon cancer cells whose expression of UDG was depleted by shRNA (Figure 1A, 1B). We then determined if the enzymatic activity of UDG is reduced in UDG depleted cells by the glycosylase activity assay. In brief, we incubated isolated nuclear extracts with a fluorescently tagged 40-mer DNA duplex that contains a U:A base pair. If the activity of UDG is intact, the uracil base will be removed, creating an abasic/apyrimidinic (AP) site. AP sites will be subsequently cleaved by the downstream BER protein AP endonuclease (APE) to generate a 23-mer band that can be visualized by gel electrophoresis (Figure 1C). As expected, purified UDG and APE enzymes efficiently removed uracil in the DNA duplex (Figure 1D, lane 3), serving as a positive control. Nuclear extracts from non-targeted scramble (shSCR)-transfected cells almost completed removed uracil bases (oligo cutting) (Figure 1D, lane 4). However, extracts from shUDG-transfected cells exhibited markedly reduced activity of removing uracil (minimal cutting) (Figure 1D, lane 5). These results confirm that UDG is the major contributor to the uracil removal from DNA in cells.

To further study the role of UDG in removing genomic uracil and/or 5-FU, we assessed the levels of uracil and 5-FU in cellular DNA after 5-FdU treatment by the AP site detection assay. Since dUTP and 5-FdUTP pools are not elevated in cancer cells cultured with standard serum in response to 5-FdU [13], we used medium containing 10% dialyzed serum in this study. We first extracted DNA from cells treated with 5-FdU, exposed the DNA to exogenous UDG to remove residual uracil and 5-FU bases, and then the newly generated AP sites were detected by a novel near infrared (NIR) cyanine-based probe that we previously synthesized and reported [30]. The results showed that the levels of AP sites in shSCR-transfected cells remained low after 5-FdU treatment even at high concentrations (Figure 1E). In contrast, DNA from shUDG-transfected cells displayed a dramatic increase in the levels of detected AP sites in a 5-FdU dose dependent manner (Figure 1E), suggesting accumulation of genomic uracil and 5-FU in UDG depleted cells.

AP sites are the common product of removal of uracil and/or 5-FU from DNA. Therefore, the AP site detection assay provides an assessment of the combined cellular levels of uracil and 5-FU but cannot distinguish which one is dominant. Since the pathways of uracil and 5-FU incorporation differ (TS inhibition leads to uracil incorporation, whereas phosphorylation of 5-FdU leads to 5-FU incorporation), the individual levels of uracil and 5-FU may determine which pathway predominantly contribute to UDG removable lesions. To address this issue, we isolated genomic DNA from cells treated with 5-FdU, incubated the DNA with purified UDG, and measured the levels of released uracil and 5-FU by LC-MS/MS. Very low levels of uracil and 5-FU were detected from shSCR-transfected cells even after treatment with high concentrations of 5-FdU (Figure 1F), indicating efficient removal of these bases from DNA by UDG. On the other hand, a significant increase of both uracil and 5-FU was detected from shUDG-transfected cells after 5-FdU treatment (Figure 1F). These data demonstrate that 5-FdU treatment leads to roughly equivalent incorporation of both uracil and 5-FU into DNA, indicating that both lesions can contribute to the genotoxicity. These results further suggest that UDG plays a major role in removing these bases and limiting such toxicity.

### Loss of UDG enhances cytotoxicity of 5-FdU in cancer cells

**Figure 2:**
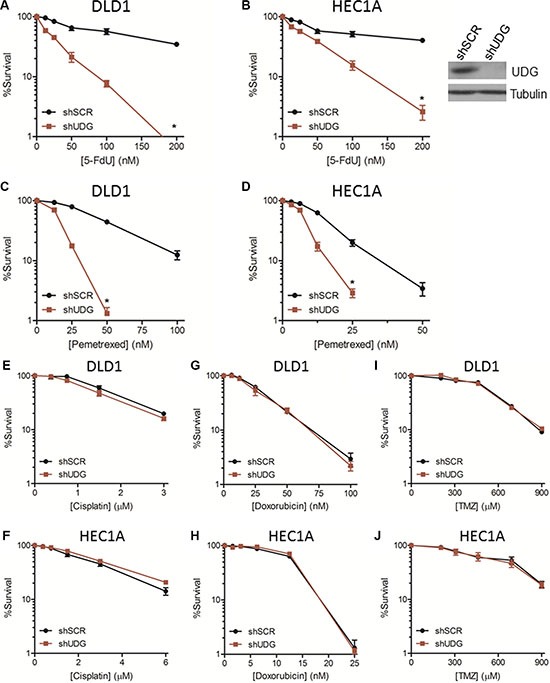
UDG depletion enhances 5-FdU sensitivity in cancer cells Colony survival assays in (**A**) DLD1 and (**B**) HEC1A shSCR and shUDG cancer cells treated with increasing doses of 5-FdU, and cell survival was measured as described in Materials and Methods. UDG expression level in HEC1A cells was determined by western blot (inset). Colony survival assays in (**C**) DLD1 and (**D**) HEC1A shSCR and shUDG cells treated with increasing doses of pemetrexed. Colony survival assays in DLD1 and HEC1A shSCR and shUDG cells treated with increasing doses of (**E**, **F**) cisplatin, (**G**, **H**) doxorubicin, or (**I**, **J**) temozolomide (TMZ). Viable colonies (> 50 cells) stained with methylene blue after 10 d of culture were counted. All survival data represent mean and SEM from at least 3 independent experiments. (*P < 0.05).

To address the role of UDG in determining the cytotoxicity of 5-FdU, we measured the cell survival of DLD1 colon cancer cells and HEC1A endometrial cancer cells in response to 5-FdU by colony survival assays. The results showed that 5-FdU caused a moderate loss of cell viability in shSCR-transfected cells at high concentrations (Figure 2A, 2B). Notably, loss of UDG highly sensitized cancer cells to 5-FdU treatment (Figure 2A, 2B). This sensitization was also observed in UDG depleted DLD1 and HEC1A cancer cells treated with pemetrexed (Figure 2C, 2D), an antifolate that can also block TS and introduce uracil incoporation into DNA. In contrast, UDG depleted DLD1 and HEC1A cells displayed no further sensitivitiy to cisplatin (Figure 2E, 2F), a crosslinking agent, doxorubicin (Figure 2G, 2H), a DNA intercalating agent, or temozolomide (Figure 2I, 2J), an alkylating agent, indicating that UDG is not involved in removing crosslinked, intercalated, or methylated nucleotides from DNA. Collectively, these data demonstrate that loss of UDG increases the sensivity of cancer cells to agents that induce uracil or 5-FU incorporation into DNA, suggesting that UDG plays an important role in determining the cell killing effect of these drugs.

### Thmidine treatment after 5-FdU exposure cannot fully rescue the enhanced cytotoxicity in UDG depleted cells due to the retention of genomic uracil and 5-FU

**Figure 3:**
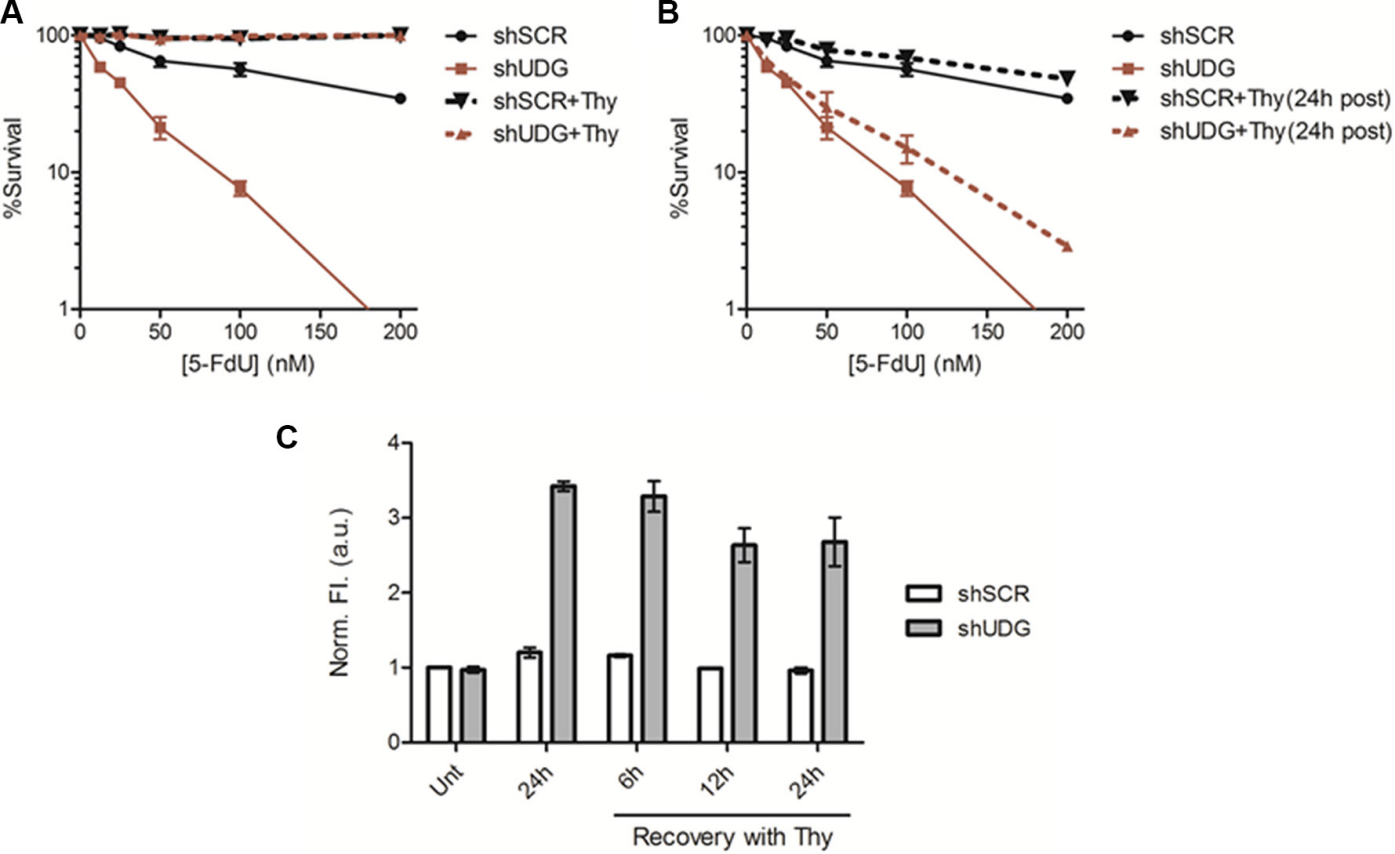
Thymidine treatment after 5-FdU exposure cannot fully rescue increased cytotoxicity in UDG depleted cells (**A**) Colony survival assay in DLD1 shSCR and shUDG cells treated with 0 to 200 nM 5-FdU alone, or supplemented with 20 μM thymidine simultaneously during 5-FdU treatment (+Thy). (**B**) Colony survival assay in DLD1 shSCR and shUDG cells treated with 0 to 200 nM 5-FdU alone, or supplemented with 20 μM thymidine 24 h after 5-FdU treatment ((+Thy (24 h post)). Data represent mean and error from at least 3 independent experiments. (*P < 0.05) (**C**) DLD1 shSCR and shUDG cells were treated with 100 nM 5-FdU for 24 h, then washed twice with PBS, and incubated in drug-free media supplemented with 20 μM thymidine (Thy) for 6, 12, or 24 h. Genomic DNA was extracted and treated *in vitro* with purified UDG. AP sites detection was performed by incubation of DNA with a cyanine-based AP site probe. Data represent mean and SD of relative fluorescence intensity normalized to the shSCR DNA without 5-FdU treatment from three independent experiments.

Thymidine deficiency has been generally considered as the main cytotoxic mechanism of TS inhibitors [1, 4–6]. However, our data suggest that it is the incoporation and the lack of removal of genomic uracil and 5-FU lesions that caused the enhanced cytotoxicity of UDG depleted cells to 5-FdU. The replenishment of thymidine should bypass the thymidine deficiency induced by 5-FdU and also reduce the incorporation of either uracil or 5-FU into DNA, a downstream effect of a shortage of thymidine pool. To test this hypothesis, we first examined the effect of simultaneous treatment of thymidine and 5-FdU (shSCR+Thy, shUDG+Thy), which was intended to completely block the thymidineless effect from the beginning. Under these conditions, there was almost no killing in either shSCR-transfected or shUDG-transfected cells (Figure 3A). However, when thymidine was replenished 24 h after 5-FdU treatment (shSCR+Thy (24 h post), shUDG+Thy (24 h post)), it barely inhibited cell death of UDG depleted cells caused by 5-FdU (Figure 3B), indicating that the enhanced killing effect by UDG depletion is due to the incorporation of uracil and 5-FU into DNA instead of the lack of thymidine. To further prove that uracil and 5-FU lesions are indeed retained in DLD1 UDG depleted cells even during recovery in the presence of thymidine, we performed the AP site detection assay in cells treated with thymidine after 24 h of 5-FdU exposure. The results showed that UDG depleted cells accumulated about three times higher the level of uracil and 5-FU than shSCR-transfected cells following 24 h of 5-FdU treatment (Figure 3C). After 24 h of 5-FdU exposure, cells were washed and placed in drug-free medium supplemented with thymidine. Notably, we observed that the uracil and 5-FU levels in UDG depleted cells remained persistent during 6, 12, and 24 h of thymidine recovery (Figure 3C). Furthermore, the retention of uracil and 5-FU during thymidine recovery following 5-FdU treatment was also detected in HEC1A UDG depleted cells (Supplementary Figure S1). Taken together, these data suggest that the enhanced cytotoxicity in UDG depleted cells is attributed to the retention of uracil and 5-FU in DNA.

### UDG depletion leads to cell cycle arrest at late G1 and early S phase by 5-FdU

**Figure 4:**
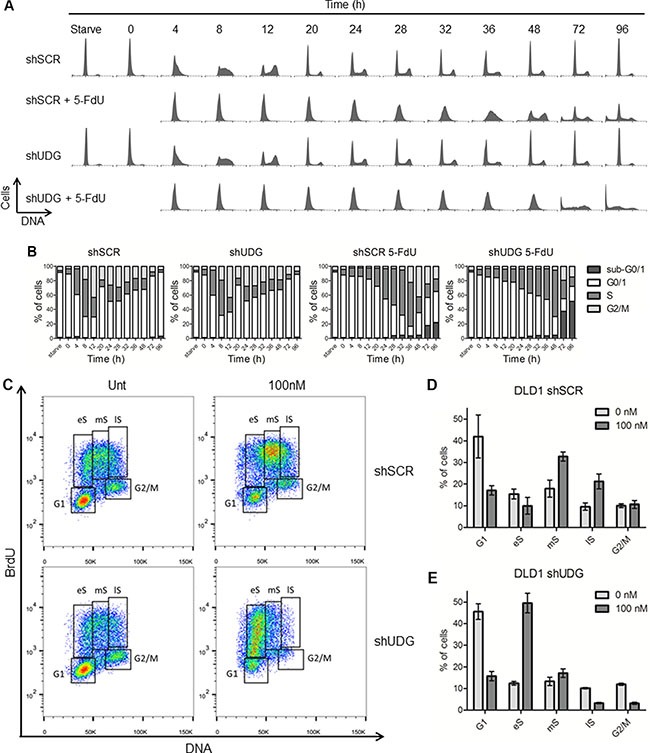
Loss of UDG induces cell cycle arrest at late G1 and early S phase by 5-FdU exposure (**A**) DLD1 shSCR and shUDG cells were synchronized at G0/G1 phase by serum starvation for two days indicated as Starve. Cell cycle and growth were resumed by releasing cells into medium containing 10% dialyzed FBS for 16 h. Cells were then exposed to 100 nM 5-FdU for indicated times (0–96 h). Cell cycle of untreated and treated cells was analyzed by PI mediated flow cytometry. (**B**) Quantification of each phases of the cell cycle for shSCR and shUDG cells from A. (**C**) Unsynchronized DLD1 shSCR and shUDG cells were untreated (Unt) or treated with 100 nM 5-FdU for 24 h and pulsed with BrdU for 45 minutes. Cells were collected, fixed and stained with anti-BrdU antibody and PI dye. Cell cycle profiles were analyzed by flow cytometry. eS = early S-phase; mS = mid-S-phase; lS = late S/G2-phase. Quantification of each phases of the cell cycle for DLD1 (**D**) shSCR and (**E**) shUDG cells from C. Data for a representative experiment that has been performed three times is shown.

Studies have shown that TS inhibition leads to S phase arrest by blocking DNA replication as a result of dTTP deficiency [31–33]. To elucidate the molecular mechanisms by which UDG regulates cellular sensitivity to 5-FdU, we monitored cell cycle progression by propidium iodide (PI) staining. DLD1 cells were synchronized at G0/G1 phase through serum starvation, resumed growth by placing in medium containing 10% dialyzed FBS for 16 h which did not result in progression through cell cycle, and then exposed to 5-FdU for an additional 0 to 96 h. In the absence of 5-FdU, both shSCR-transfected and shUDG-transfected cells progressed similarly through S and G2/M phases by 8 and 12 h, respectively (Figure 4A, 4B), indicating that UDG depletion did not affect normal cell cycle progression. As expected, 5-FdU slowed the progression of shSCR-transfected cells through S phase by 36 h, and cells entered the next cell cycle by 48 h with a relatively small portion of cells at sub-G1 phase (Figure 4A, 4B). However, 5-FdU treatment triggered a strong cell cycle arrest of UDG depleted cells at late G1 and early S phase which lasted for 48 h and later displayed a chaotic cell cycle distribution pattern at 72 h and 96 h with substantially increased sub-G1 population (Figure 4A, 4B).

To confirm the cell cycle arrest results, we monitored the S phase population of unsynchronized cells by BrdU and PI co-staining in DLD1 cancer cells. Consistently, we observed S phase arrest especially at middle and late S phase in shSCR-transfected cells as a result of TS inhibition after 24 h of 5-FdU exposure (Figure 4C, 4D). In contrast, DLD1 shUDG-transfected cells were arrested at late G1 and early S phase following 24 h of 5-FdU exposure (Figure 4C, 4E). In addition, the G1/S phase arrest was also confirmed in HEC1A UDG depleted cells (Supplementary Figure S2). Together, these findings implicate that loss of UDG affects cell cycle progression at early S phase in response to continuous 5-FdU exposure, likely due to the accumulation of uracil and 5-FU in genomic DNA that blocks DNA replication.

### Loss of UDG inhibits DNA replication progression in response to 5-FdU treatment

**Figure 5:**
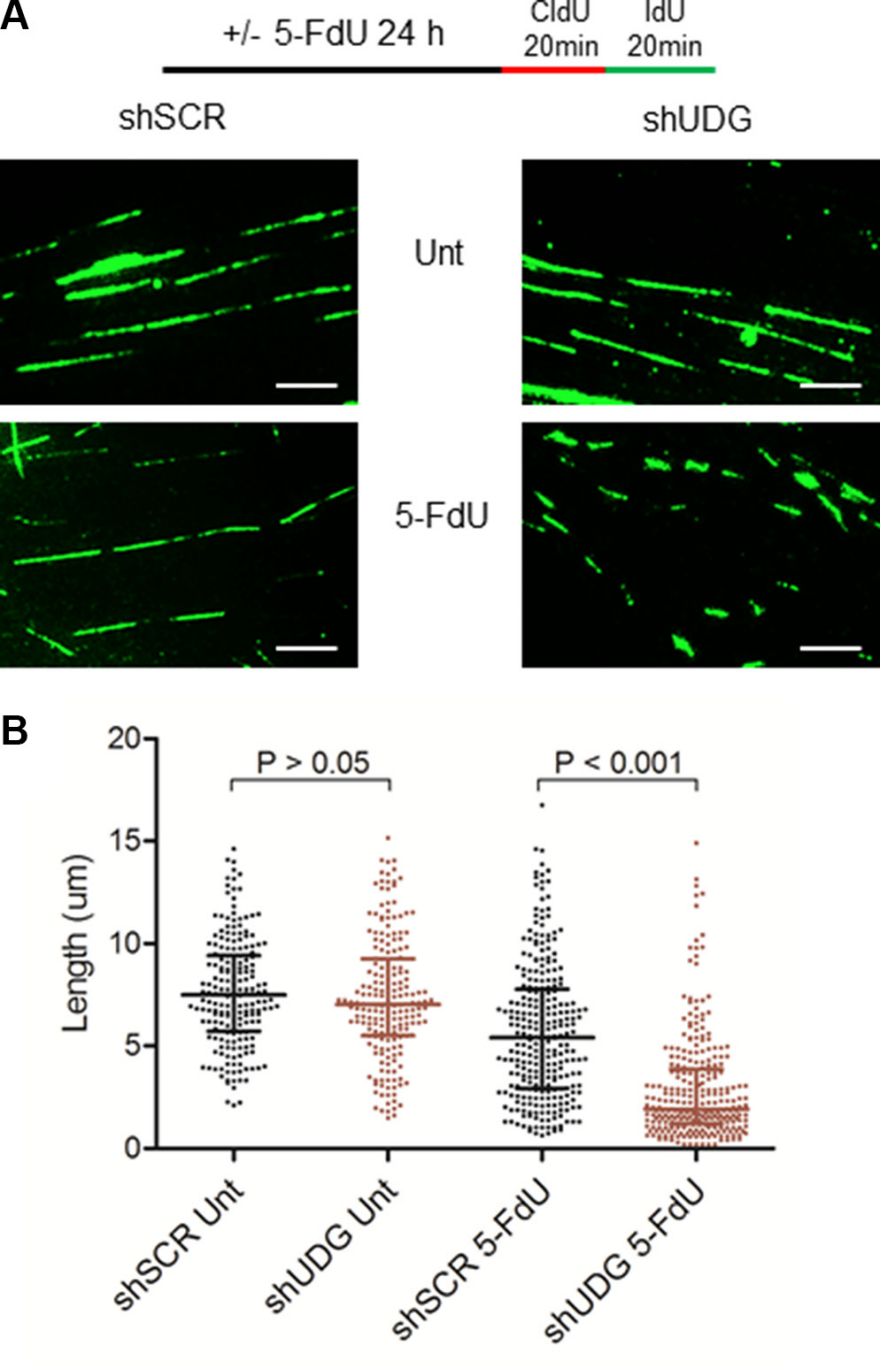
UDG depletion inhibits replication fork progression following 5-FdU treatment (**A**) DLD1 shSCR and shUDG cells were untreated (Unt) or treated with 100 nM 5-FdU for 24 h, washed, pulsed with CIdU and IdU sequentially for 20 minutes. Cells were lysed and DNA fragments were spread on the slide. The fixed samples were stained with anti-CIdU and anti-IdU antibodies. DNA fibers were visualized on fluorescence microscope (100X oil lens). (Scale bar: 5 μm) (**B**) Quantification of the DNA fiber length. The statistical analysis of DNA fiber length across the populations analyzed (*n* > 200 fibers per population) is shown as a scatter plot with medians and the interquartile ranges. To monitor the replication progression speed, we only counted the IdU track as it represents ongoing replication length.

To directly investigate the mechanism by which 5-FdU arrests UDG depleted cells at G1/S phase, we monitored replication fork progression by DNA fiber analysis [34]. Following 24 h 5-FdU treatment, DLD1 cells were sequentially pulsed with halogenated nucleotides chlorodeoxyuridine (CldU) and iododeoxyuridine (IdU) for 20 minutes (Figure 5A). DNA fibers stained with both CldU (red, not shown) and IdU (green) were included in the following analysis. To assess the impact on DNA replication progression, we measured the track length of IdU as it represents the ongoing replication fork. In the absence of 5-FdU, the mean fiber length for both shSCR- and shUDG-transfected cells was around 7.5 μm (Figure 5B). Following 24 h 5-FdU exposure, the mean fiber length of nascent DNA strands reduced by 23% to 5.7 μm in shSCR-transfected cells, consistent with the temporal S phase arrest results (Figure 4). Strikingly, UDG depleted cells displayed significantly shorter fiber track with the mean value at 2.8 μm, representing a 63% reduction (Figure 5B), consistent with the prolonged G1/S arrest. These results illustrate that loss of UDG inhibits DNA replication in response to 5-FdU by severely reducing the elongation of nascent DNA strands.

### DNA damage persists in UDG depleted cells and is not due to apoptosis by 5-FdU treatment

**Figure 6:**
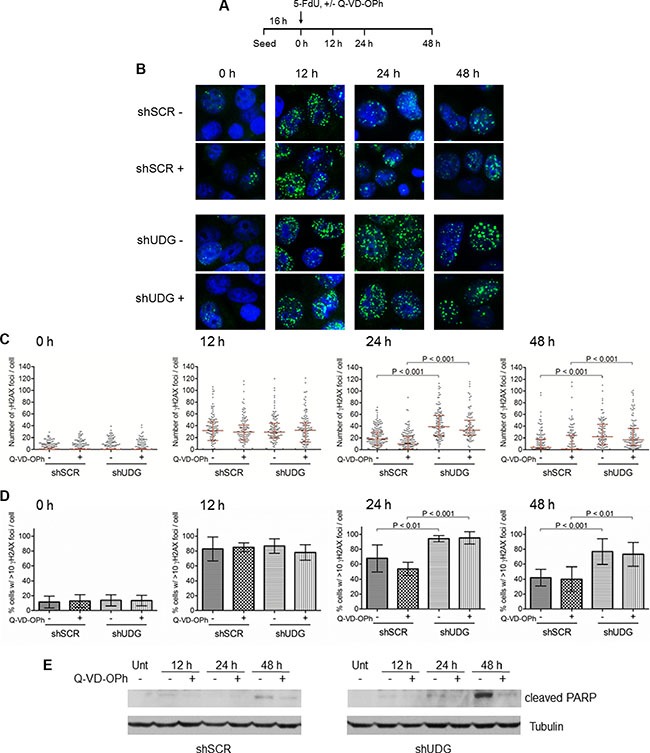
DNA damage accumulates in UDG depleted cells in a caspase independent manner (**A**) Schematic diagram of the treatment of DLD1 cells with 5-FdU in the presence or absence (+/−) of 10 μM caspase inhibitor Q-VD-OPh at indicated time points. (**B**) DLD1 shSCR and shUDG cells were treated with 50 nM 5-FdU for 12, 24, and 48 h with (+) and without (−) 10 μM Q-VD-OPh. Cells were fixed and stained with anti-γH2AX antibodies. γH2AX foci was visualized on a fluorescence microscope. (**C**) Quantification of the number of γH2AX foci per cell for 0, 12, 24, and 48 h of 5-FdU treatment in the presence (+) or absence (−) of Q-VD-OPh. The statistical analysis of γH2AX foci per cell across the populations analyzed (*n*> 100 cells per population) is shown as a scatter plot with medians and the interquartile ranges. (**D**) Quantification of the percentage of cells with >10 γH2AX foci per cell for 0, 12, 24, and 48 h of 5-FdU treatment. Statistical analysis was performed as in C. (**E**) In parallel samples from B, the expression level of cleaved PARP was analyzed for cells untreated (Unt) or treated with 50 nM 5-FdU for 12, 24, and 48 h in the presence (+) or absence (−) of 10 μM caspase inhibitor Q-VD-OPh.

The dramatic increase in sub-G1 population in UDG depleted cells by 5-FdU indicates that these cells are undergoing apoptotic cell death. However, what caused the cell death remains unclear. Prolonged replication fork stalling due to dNTP imbalance can lead to fork collapse and the generation of DNA double strand breaks (DSBs) [35, 36], a highly mutagenic and toxic form of DNA damage. To understand if UDG depleted cells accumulate DNA damage by 5-FdU treatment, we performed immunostaining to assess the generation of DSBs using specific antibodies to detect foci formation of the phosphorylated histone variant H2AX (γH2AX), a marker of DSBs (Figure 6A). In DLD1 shSCR-transfected cells, 5-FdU caused the maximal increase in the level of DSBs and the percentage of cells with over 10 foci by 12 h of treatment, which then gradually declined despite the presence of 5-FdU (Figure 6B–6D), indicating cells expressing UDG are able to repair DNA damage even in the presence of 5-FdU. On the other hand, both the foci number and the percentage of cells with over 10 foci remained persistent during 5-FdU exposure in DLD1 UDG depleted cells (Figure 6B–6D), suggesting sustained DNA damage in the absence of UDG. Consistently, in HEC1A shSCR-transfected cells, the maximal level of DSBs and the percentage of cells with over 10 foci were detected at 48 h of 5-FdU treatment, which then reduced at 72 h and 96 h of treatment (Supplementary Figure S3A–S3D). However, in HEC1A UDG depleted cells, the foci number and the precentage of cells with over 10 foci remained high during 5-FdU exposure (Supplementary Figure S3A–S3D).

Caspase activation during apoptosis also leads to DNA fragmentation and damage [37, 38]. Therefore, to prove that the formation of DNA damage is the cause, but not the consequence of cell death induced by 5-FdU, we monitored γH2AX foci in both DLD1 and HEC1A cells in the presence or absence of a broad-spectrum caspase inhibitor Q-VD-OPh [39–41]. If DNA damage were the consequence of caspase activation, then we would expect that the caspase inhibitor should abolish γH2AX foci formation. However, we observed that the number of γH2AX foci and the percentage of γH2AX positive cells were essentially the same between Q-VD-OPh treated and non-treated shSCR-transfected or shUDG-transfected cells (Figure 6B–6D, and Supplementary Figure S3B–S3D). These data strongly suggest that the increased DNA damage induced by 5-FdU is not the result of caspase activation. To prove that the caspase inhibitor indeed blocked the apoptotic signaling, we examined the expression of cleaved PARP, a marker of apoptosis, in parallel samples. We found that cleaved PARP by 5-FdU treatment was almost completely blocked by the Q-VD-OPh treatment in both shSCR-transfected and shUDG-transfected cells (Figure 6E, and Supplementary Figure S3E). In addition, the appearance of cleaved PARP in DLD1 and HEC1A cells was not evident until after 24 h and 96h of 5-FdU treatment, respectively (Figure 6E, and Supplementary Figure S3E), whereas DSBs formation was readily detected at 12 h and 48 h of treatment (Figure 6B–6D, and Supplementary Figure S3B–S3D). Collectively, these results demonstrate that the formation of DSBs precedes the apoptosis signaling caused by 5-FdU in UDG depleted cells, suggesting that DNA damage is the cause of cell death.

## DISCUSSION

5-FdU metabolite blocks TS, causing nucleotide pool imbalance, which favors uracil and 5-FU incorporation into DNA. Previously, studies have demonstrated elevated genomic uracil levels in UDG deficient non-cancer cells [21, 22], and increased levels of genomic 5-FU in UDG depleted cancer cell following 5-FdU exposure [23]. To the best of our knowledge, the present study is the first to illustrate the individual levels of incorporated uracil and 5-FU simultaneously in response to 5-FdU. Using both the AP site detection assay and mass spectrometry analysis, we found similar levels of uracil and 5-FU incorporated into cellular DNA following 5-FdU exposure in UDG depleted cancer cells. Collectively, these studies indicate that the absence of uracil and 5-FU in UDG competent cells reflects a predominant role of UDG in preventing abnormal base accumulation in genomic DNA.

Previous studies reported inconsistent roles of UDG in determining the sensitivity of TS inhibitors and, of note, most of these studies were conducted in non-cancer cells [13, 20–22, 24]. In human colon tumors, the UDG activity has been reported to be significantly higher than in normal bowel tissues [29], suggesting UDG as a potential biomarker to predict 5-FdU resistance in colon cancer and also a potential target for inhibition. The study of Huehl et al. (2016) firstly showed that loss of UDG sensitized cancer cells to 5-FdU [19], which is consistent with our findings. Here we further demonstrate that this sensitization can only be rescued when thymidine was added simultaneously, but not after 5-FdU treatment, indicating that the cytotoxicity was mainly caused by the accumulation of uracil and 5-FU bases in DNA in the absence of UDG. We previously observed sensitization of UDG depleted cancer cells to another TS inhibitor, pemetrexed, suggesting that the genomic uracil incorporation alone is toxic to cells [17]. Although Huehl et al. (2016) suggested that incorporation of 5-FU into DNA played a more important role than uracil in contributing to 5-FdU-induced cell death [19], they did not measure incorporated uracil levels and therefore may have underestimated the contribution of uracil incorporation to 5-FdU's cytotoxicity and DNA replication fork disruption. Nevertheless, these findings together demonstrate that loss of UDG in cancer cells enhances the killing effect of 5-FdU, a TS inhibitor through the incorporation of the abnormal bases uracil and 5-FU into DNA.

In addition to UDG that acts on uracil and 5-FU in DNA, TDG was also reported to preferentially excise uracil and 5-FU that is mispaired with guanine [42–44]. However, loss of TDG that confers 5-FU resistance has been observed in MEF cells in a manner different from that of UDG in our study [27]. The excision of uracil and 5-FU from DNA by TDG was thought to precipitate the cytotoxicity of 5-FU due to the slow dissociation of TDG from AP sites, therefore blocking the downstream repair pathway [13, 27]. Importantly, it has been reported previously that TDG is absent from S phase cells, while UDG expression, on the contrary, is highly induced in S phase [45, 46]. Our findings revealed that UDG depletion leads to accumulation of DNA lesions including both uracil and 5-FU incorporation during S phase in response to 5-FdU treatment. Once the cells exit S phase where TDG is expressed, these lesions will be recognized by TDG that slows down the repair process and contributes to additional cytotoxicity. Under these assumptions, TDG would synergize with the inhibition of UDG following 5-FdU exposure.

Knowing that the presence of UDG significantly compromised the cytotoxic effect of 5-FdU by limiting the existence of DNA lesions of uracil and 5-FU, we sought to understand how these lesions led to cytotoxicity in cancer cells. We found that genomic uracil and 5-FU incorporation, which is a downstream effect of TS inhibition by 5-FdU, induced cell cycle arrest at late G1 and early S phase, indicating replication fork stalling at early phases of DNA synthesis. This severely stalled or collapsed DNA replication was confirmed and quantified via the DNA fiber analysis. We previously reported that uracilated DNA induced by pemetrexed treatment arrested UDG depleted DLD1 cells at S phase, which is in agreement with the current findings [17]. However, 5-FdU induced more profound DNA replication arrest than pemetrexed after UDG depletion. We propose two possibilities to explain these differences. First, low doses of pemetrexed were used in the previous study, which likely led to less production of dUTP than 5-FdU at the doses used herein. Second, while pemetrexed primarily induces dUTP production, 5-FdU leads to the generation of both dUTP and 5-FdUTP.

Activation of homologous recombination-induced DNA damage repair in response to TS inhibition promotes cell survival [47, 48], which could explain the disappearance of DNA damage of shSCR-transfected cells even in the presence of 5-FdU. However, loss of UDG induces accumulation of significant amounts of abnormal bases in genomic DNA followed by DNA replication arrest, which consequently leads to the generation of numerous DSBs that are likely beyond the cell's repair capability. As a result, UDG depleted cells displayed continuous DNA damage. Although numerous studies have indicated activation of apoptosis following exposure to TS inhibitors [49–51], our results suggested that it is DNA damage that induces cell death, but not the other way around. The time course studies of γH2AX foci formation in the presence or absence of caspase inhibitor confirmed this idea, in which DNA damage precedes the activation of cell death signaling pathway in cells depleted of UDG. These results strongly support the idea that loss of UDG significantly enhances the cell killing effect of 5-FdU through the generation of excessive DNA damage.

While uracil and/or 5-FU incorporation into DNA has been recognized for decades, this is one of the few studies to define its mechanism of toxicity in the absence of removal by UDG. Further, from a clinical point of view, our studies clarify the utility of targeting UDG to improve the anti-cancer efficacy of commonly used chemotherapeutic agents.

## MATERIALS AND METHODS

### Cell lines and drugs

DLD1 colon cancer cells were purchased from American Type Culture Collection, and HEC1A cells were a gift from Dr. Sanford Markowitz at Case Western Reserve University. Cells were maintained in growth medium DMEM supplemented with 10% dialyzed fetal bovine serum containing penicillin and streptomycin. Cells were incubated at 37°C in a humidified atmosphere of 5% CO_2_. Drugs and chemicals used in this study are: 5-fluorodeoxyuridine (Sigma Aldrich), thymidine (Sigma Aldrich), pemetrexed (LC laboratories), temozolomide (Ochem Inc), cisplatin and doxorubicin (kindly provided by Dr. John Pink at Case Western Reserve University).

### Lentiviral shRNA knockdown

UDG knockdown was performed via shRNA transduction with validated clone from Sigma-Aldrich. The ID of UDG shRNA clone is NM_003362.2-656s21c1. A non-targeted scramble control shRNA clone (Sigma-Aldrich) was also used. Transfection of shRNA clones was performed according to manufacturer's specifications from Lipofectamine 2000 (Invitrogen). Lentiviral particles were produced *via* HEK293 cells, and targeted cells were infected and selected with puromycin. The stable UDG knockdown levels were verified for q-PCR and western blot analysis.

### Glycosylase activity assay

UDG activity was determined by using a green emitting Alexa 532 labeled 40-mer duplex DNA containing a U:A base pair that was synthesized by IDT with the sequence:

5′-TCCTGGGTGACAAAGCUAAACACTGTCTC CAAAAAAAATT [Alexa]-3′

3′-AGGACCCACTGTTTCGATTTGTGACAGAG GTTTTTTTTAA-5′

For the reaction, 5 pmol (10 μL) diluted DNA aliquots were incubated with either purified enzymes UDG and APE (New England Biolabs) sequentially or 10 μg nuclear extracts isolated from cells at 37°C for 20 minutes. Nuclear extracts were prepared by using the NucBuster isolation procedure (EMD Bioscience Calbiochem). Reaction products were resolved in the dark by electrophoresis on 20% denaturing polyacrylamide gels (5.3 g urea, 5.0 mL 40% acrylamide, 2.3 mL 5X TBE buffer, 200 μL 10% APS, and 20 μL TEMED). Gels were visualized by a Typhoon Tri + Variable Mode Imager (Amersham Biosciences).

### Apyrimidinic (AP) site detection

The amount of cellular AP sites was assessed as we previously described by using a NIR cyanine-based AP site probe [30]. Briefly, following 5-FdU exposure, genomic DNA was obtained from phenol-chloroform extraction, dissolved in 1X UDG reaction buffer (20 mM Tris-HCl, 1 mM EDTA and 1 mM dithiothreitol, pH 8.0), and incubated with either the UDG enzyme (1 μL, 5 units) or 1 μL UDG storage buffer (10 mM Tris-HCl, 50 mM KCl, 1 mM DTT, 0.1 mM EDTA, 0.1 mg/ml BSA, 50% Glycerol, pH 7.4) as a vehicle control at 37°C for 1 h. After the reaction, AP site probe with a final concentration of 25 μM was added and incubated at 37°C for 1 h. Following incubation, extracted DNA was precipitated, and the supernatant was discarded. DNA pellets were resuspended in H_2_O, and DNA concentrations were measured and adjusted. The fluorescence intensities of each sample were analyzed with 760 nm excitation and emission scan of 790–847 nm.

### Quantitative determination of uracil and 5-FU incorporated in cellular DNA by LC-MS/MS

Genomic DNA was extracted from cells treated with 5-FdU *via* phenol-chloroform mixture. 80 μg of DNA sample was dissolved in 1X UDG reaction buffer (20 mM Tris-HCl, 1 mM EDTA and 1 mM dithiothreitol, pH 8.0) and incubated with UDG enzyme (1 μL, 5 units) for 1 h at 37°C. For LC-MS/MS analysis of DNA-incorporated uracil and 5-FU, 75 μL of the enzyme reaction mixture was obtained, and uracil-1,3-^15^N_2_ was used as the internal standard (Sigma-Aldrich). All uracil and 5-FU standards, internal standard, and QC samples were prepared in 1X UDG reaction buffer. The separation of analytes were achieved by a Shimadzu LC-20AD HPLC system with a Shimadzu SIL-20AC autosampler (Shimadzu) on a Waters Xbridge HILIC pre-column (2.1 × 10 mm, 3.5 μm) and a Xbridge HILIC column (2.1 × 100 mm, 3.5 μm) (Waters Corporation) using a mobile phase consisting of 87.5% acetonitrile and 12.5% 10 mM ammonium formate at a flow rate of 0.200 mL/min. Quantitation of the analytes was accomplished by a AB Sciex API 3200 triple quadrupole tandem mass spectrometer (AB Sciex), which was operated in the negative multiple-reaction-monitoring (MRM) mode with mass transitions of m/z 110.8 > 42.0 for uracil, m/z 112.9 > 43.0 for uracil-1,3-^15^N_2_ and m/z 129.0 > 42.0 for 5-FU. This method has lower limits of quantitation of 2.50 ng/mL and linear calibration ranges up to 500 ng/mL for both uracil and 5-FU with a sample injection volume of 15 μL, as well as a total analysis time of 6 min.

### Colony survival assay

DLD1 (200 cells/well) or HEC1A (300 cells/well) cells were plated in 6-well culture dishes and allowed to adhere for 16 h. Cells were treated with drug for 24 h, gently washed twice with 1X PBS, and incubated with fresh media for at least 10 days to allow individual colonies to form. Colonies were stained with methylene blue, and only colonies containing ≥ 50 cells were counted. The percentage of survival was determined relative to untreated control averaged over 3 independent experiments.

### Cell cycle and bromo-deoxyuridine (BrdU)/PI labeling analysis

For cell cycle analysis, DLD1 cells were synchronize by serum starvation for 48 h and released in fresh media for 16 h. The cells were then treated with 100 nM 5-FdU for 4, 8, 12, 20, 24, 28, 32, 36, 48, 72, and 96 h. At each time point, cells were harvested and fixed with methanol. Fixed cells were incubated with DNase-free RNaseA (Roche) and stained with 50 μg/mL PI solution (Sigma-Aldrich). For BrdU/PI labeling analysis, cells were treated with 100 nM 5-FdU for 24 h and pulsed with 10 μM BrdU (BD Biosciences Pharmingen, BrdU Flow Kit) for 45 minutes before collecting cells. According to manufacturer's instructions from BD Biosciences Pharmingen, cells were fixed, treated with DNAse for 1 h at 37°C, stained with FITC anti-BrdU for 20 minutes, and incubated with PI staining solution (50 μg/mL PI, 10 mM Tris-HCl pH 7.5, 5 mM MgCl2, 10 μg/mL DNase-free RNaseA) for 30 minutes at 37°C. For both assays, cells were analyzed on a BD LSRII instrument.

### DNA fiber assays

DNA fiber analysis was performed as described [34]. Cells treated with 100 nM 5-FdU for 24 h were pulse-labeled with 100 μM chlorodeoxyuridine (CIdU) for 20 minutes, washed with PBS, and 25 μM Iododeoxyuridine (IdU) for 20 minutes. Cells were collected in PBS, and 2.5 μL of cell suspension was dropped on glass slide. 7.5 μL of lysis buffer (0.5% SDS, 200 mM Tris-HCl pH 7.4, 50 mM EDTA) was dropped on the cell suspension and lysis for 10 minutes. Slides were then tilted at 15° to spread the suspension and placed horizontally to allow air-dry. After drying, slides were fixed in 3:1 methanol:acetic acid for 15 minutes, washed with water, and placed at −20°C overnight. Slides were then treated with 2.5 M HCl for 1 h, washed with PBS containing 0.1% Tween-20, washed twice with PBS, blocked in PBS containing 5% BSA and 0.1% Tween 20 for 20 minutes, and rinsed with PBS three times. After washing, 100 μL primary antibodies: mouse anti-BrdU/IdU (Becton Dickinson, 1:100) and rat anti-BrdU/CIdU (AbD Serotec, 1:400) diluted in PBS containing 5% BSA and 0.1% were added to incubate in a humid chamber for 4–6 h. After incubation, slides were washed with PBS three times, incubated with secondary fluorescent antibodies: sheep anti-mouse Alexa Fluor 488 (Life technologies) and donkey anti-rat Alexa Fluor 594 (Life technologies) diluted in PBS containing 5% BSA for 1 h. Slides were washed with PBS three times and mounted with Vectashield mounting medium. Image acquisition was performed on a Leica laser microscope. DNA fiber length was measured by using ImageJ software (NCI/NIH).

### Immunofluorescence staining

Cells cultured on glass coverslips were treated with 5-FdU in the presence or absence of 10 μM caspase inhibitor Q-VD-OPH (BioVision Inc). Cells were fixed in 3.7% formaldehyde for 10 minutes, blocked with PBS containing 10% FBS and 0.1% Triton X-100 for 20 minutes, washed with PBS three times, and incubated with primary anti-γH2AX antibody (Millipore, dilution: 1:150) in PBS containing 0.1% Triton X-100 at 4°C overnight. The cells were then washed with PBS three times, incubated with secondary antibodies (Alexa Fluor 594, Life Technologies; dilution: 1:400) in PBS containing 0.1% Triton X-100 for 1 h, and washed with PBS three times. The slides were mounted with antifade solution with DAPI (Cell Signaling) and visualized on a Leica laser microscope.

### Western blots and qPCR

Western blots were performed as described [52]. Antibodies used were as follows: Anti-UDG (FL-313) (Santa Cruz Biotechnology), anti-Cleaved PARP (Asp214)(19F4) (Cell Signaling), and anti-α-Tubulin (Calbiochem). For quantitative RT-PCR, total RNA from cells was extracted using RNeasy Plus Mini Kit (Qiagen), and cDNA synthesis was carried out by using SuperScript III First Strand Kit (Life Technologies). Q-PCR was performed with validated TaqMAN MGB FAMTM dye labeled probes (Applied Biosystems) for UDG on an ABI 7500 Fast Real-time PCR System (Applied Biosystems). β-Actin was used as an endogenous control, and relative gene expression was calculated as 2^−ΔΔCt^.

### Statistics

Statistical significance between two treatment groups was determined by unpaired 2-tailed student's *t* test. Significance was assigned for a *P-value* < 0.05. Standard software GraphPad Prism (San Diego, CA, USA) and Excel 2013 (Microsoft Corp., Redmond, WA) were used for all statistical analysis.

## SUPPLEMENTARY MATERIALS FIGURES


