# Supplementary material for: Inhibition of uracil DNA glycosylase sensitizes cancer cells to 5-fluorodeoxyuridine through replication fork collapse-induced DNA damage
Source: Oncotarget. 2016 Aug 9;7(37):59299–313. doi: 10.18632/oncotarget.11151 (PMC5312313; doi:10.18632/oncotarget.11151)
Supplement: Supplementary file 1 [file oncotarget-07-59299-s001.pdf]

Inhibition of uracil DNA glycosylase sensitizes cancer cells to 5-fluorodeoxyuridine through replication fork collapse-induced DNA damage

Supplementary Materials

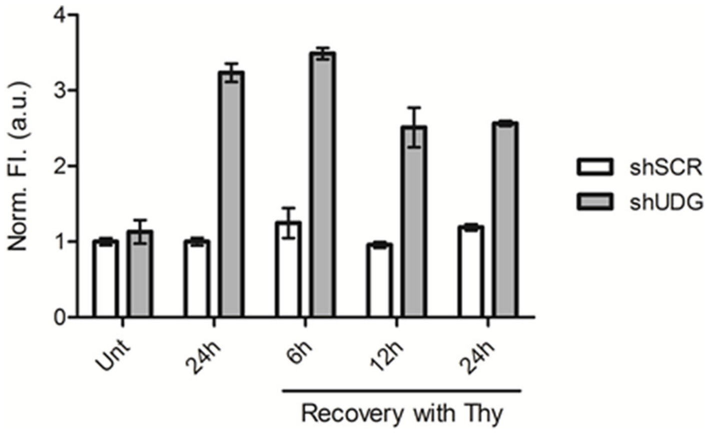

Supplementary Figure S1: Retention of uracil and 5-FU in HEC1A UDG depleted cells during thymidine recovery following 5-FdU exposure. HEC1A shSCR and shUDG cells were treated with 100 nM 5-FdU for 24 h, then washed twice with PBS, and incubated in drug-free media supplemented with 20 µM thymidine (Thy) for 6, 12, or 24 h. Genomic DNA was extracted and treated *in vitro* with purified UDG. AP sites detection was performed by incubation of DNA with a cyanine-based AP site probe. Data represent mean and SD of relative fluorescence intensity normalized to the shSCR DNA without 5-FdU treatment from three replicates.

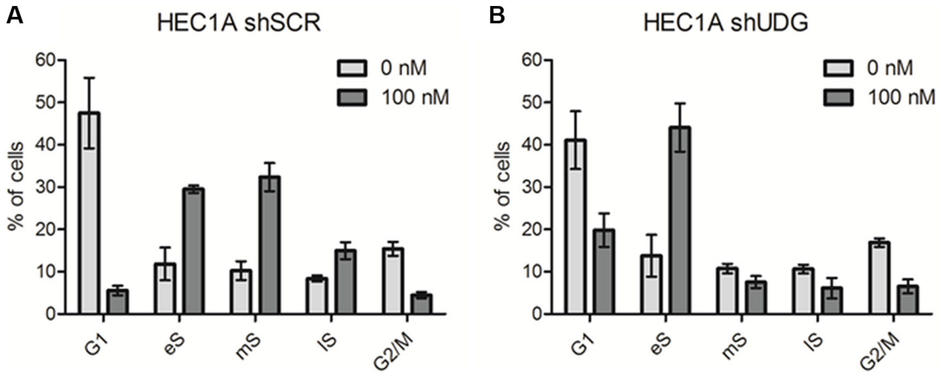

Supplementary Figure S2: Loss of UDG induces HEC1A cell cycle arrest at late G1 and early S phase following 5-FdU exposure. Quantification of each phases of the cell cycle for unsynchronized HEC1A (**A**) shSCR and (**B**) shUDG cells untreated (Unt) or treated with 100 nM 5-FdU for 24 h. After treatment, cells were pulsed with BrdU for 45 minutes, fixed, and stained with anti-BrdU antibody and PI dye. Cell cycle profiles were analyzed by flow cytometry. eS = early S-phase; mS = mid-S-phase; lS = late S/G2-phase. Data represent mean and SD from 3 independent experiments.

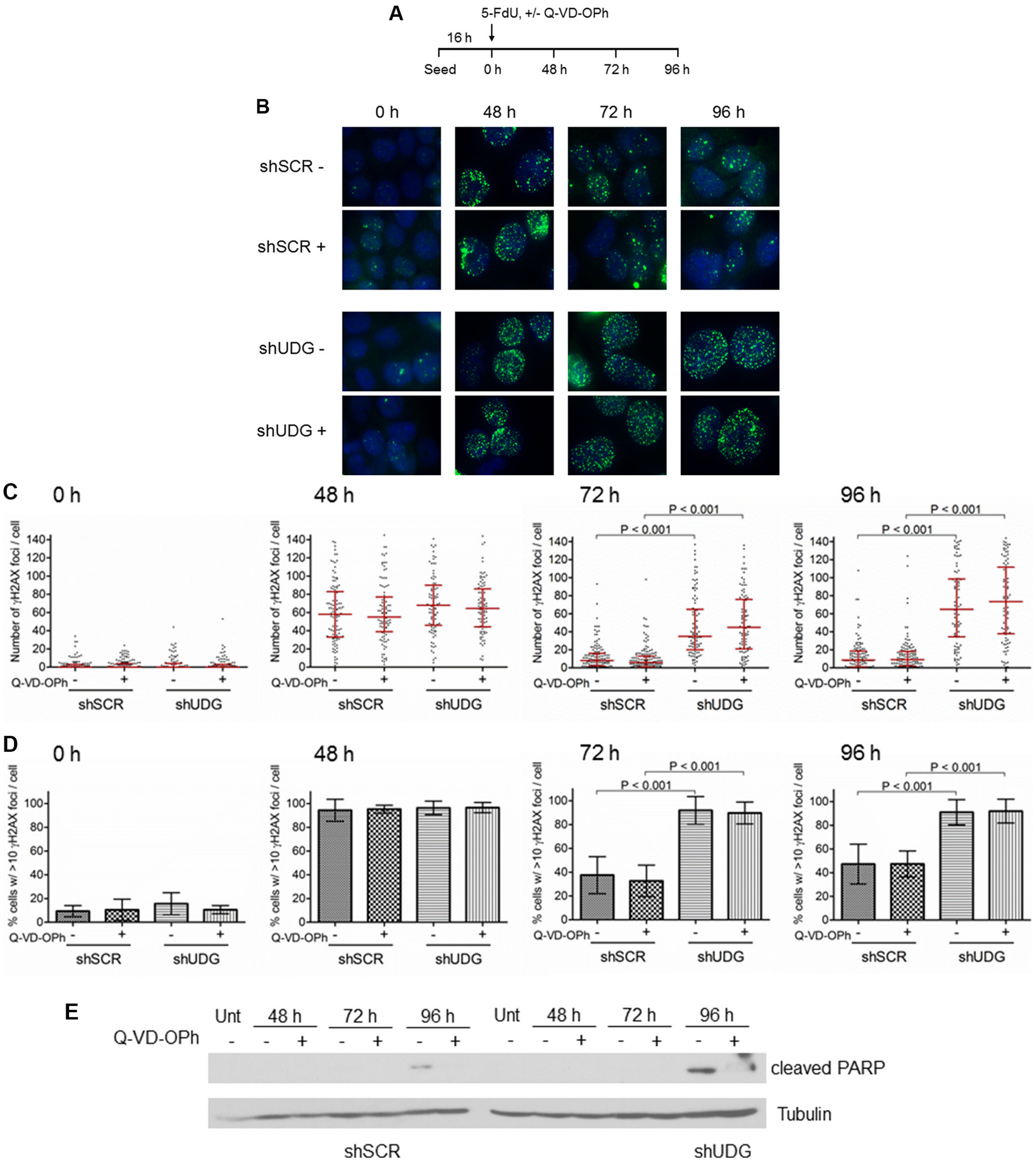

Supplementary Figure S3: DNA damage accumulates in HEC1A UDG depleted cells in a caspase independent manner. (**A**) Schematic diagram of the treatment of HEC1A cells with 5-FdU in the presence or absence (+/−) of 10 µM caspase inhibitor Q-VD-OPh at indicated time points. (**B**) HEC1A shSCR and shUDG cells were treated with 50 nM 5-FdU for 48, 72, and 96 h with (+) and without (−) 10 µM Q-VD-OPh. Cells were fixed and stained with anti-γH2AX antibodies. γH2AX foci was visualized on a fluorescence microscope. (**C**) Quantification of the number of γH2AX foci per cell for 0, 48, 72, and 96 h of 5-FdU treatment in the presence (+) or absence (−) of Q-VD-OPh. The statistical analysis of γH2AX foci per cell across the populations analyzed (*n* > 100 cells per population) is shown as a scatter plot with medians and the interquartile ranges. (**D**) Quantification of the percentage of cells with >10 γH2AX foci per cell for 0, 48, 72, and 96 h of 5-FdU treatment. Statistical analysis was performed as in C. (**E**) In parallel samples from ***B***, the expression level of cleaved PARP was analyzed for cells untreated (Unt) or treated with 50 nM 5-FdU for 48, 72, and 96 h in the presence (+) or absence (−) of 10 µM caspase inhibitor Q-VD-OPh.
